# Linking Influenza Virus Tissue Tropism to Population-Level Reproductive Fitness

**DOI:** 10.1371/journal.pone.0043115

**Published:** 2012-08-28

**Authors:** Leslie A. Reperant, Thijs Kuiken, Bryan T. Grenfell, Albert D. M. E. Osterhaus, Andrew P. Dobson

**Affiliations:** 1 Department of Virology, Erasmus Medical Centre, Rotterdam, The Netherlands; 2 Department of Ecology and Evolutionary Biology, Princeton University, Princeton, New Jersey, United States of America; 3 Fogarty International Center, National Institutes of Health, Bethesda, Maryland, United States of America; University of Texas Medical Branch, United States of America

## Abstract

Influenza virus tissue tropism defines the host cells and tissues that support viral replication and contributes to determining which regions of the respiratory tract are infected in humans. The location of influenza virus infection along the respiratory tract is a key determinant of virus pathogenicity and transmissibility, which are at the basis of influenza burdens in the human population. As the pathogenicity and transmissibility of influenza virus ultimately determine its reproductive fitness at the population level, strong selective pressures will shape influenza virus tissue tropisms that maximize fitness. At present, the relationships between influenza virus tissue tropism within hosts and reproductive fitness at the population level are poorly understood. The selective pressures and constraints that shape tissue tropism and thereby influence the location of influenza virus infection along the respiratory tract are not well characterized. We use mathematical models that link within-host infection dynamics in a spatially-structured human respiratory tract to between-host transmission dynamics, with the aim of characterizing the possible selective pressures on influenza virus tissue tropism. The results indicate that spatial heterogeneities in virus clearance, virus pathogenicity or both, resulting from the unique structure of the respiratory tract, may drive optimal receptor binding affinity–that maximizes influenza virus reproductive fitness at the population level–towards sialic acids with α2,6 linkage to galactose. The expanding cell pool deeper down the respiratory tract, in association with lower clearance rates, may result in optimal infectivity rates–that likewise maximize influenza virus reproductive fitness at the population level–to exhibit a decreasing trend towards deeper regions of the respiratory tract. Lastly, pre-existing immunity may drive influenza virus tissue tropism towards upper regions of the respiratory tract. The proposed framework provides a new template for the cross-scale study of influenza virus evolutionary and epidemiological dynamics in humans.

## Introduction

Seasonal influenza A annually causes up to a billion cases and up to half-a-million deaths worldwide, leading to considerable economic losses [1]. Influenza burdens can be increased greatly during pandemics, which are triggered by the introduction of novel influenza A viruses, typically from animal reservoirs, into the human population. Although rare events, past pandemics have each resulted in up to 50 million deaths worldwide [2].

Influenza burdens are a result of disease severity in individual hosts and the size of epidemic or pandemic waves at the population level. Influenza virus tissue tropism defines host cells and tissues that support viral replication, and governs at least partly which regions of the respiratory tract are infected in humans. The location of infection along the human respiratory tract is an essential determinant of virus pathogenicity and transmissibility [3]–[5], which are at the basis of influenza burdens. Pathogenicity typically increases as infection is located deeper down the respiratory tract because of the delicate nature and vital function of deeper airways and alveoli [3]. Conversely, transmissibility appears favoured with infection located higher up [4]–[9]. Avian influenza viruses that predominantly infect deeper regions of the respiratory tract (DRRT, i.e., bronchioles and alveoli) do not efficiently transmit among humans. Furthermore, genetic mutations in influenza virus genome that result in reduced tropism for upper regions of the respiratory tract (URRT, i.e., nose, trachea and bronchi) impair or abolish virus transmissibility in animal models [9]–[12], while genetic mutations that enhance URRT tropism can restore transmissibility [10], [12], [13].

Pathogenicity and transmissibility primarily determine influenza virus population-level fitness, usually measured by the basic reproductive number, R_0_, and defined by the number of secondary cases arising from one infected individual in a susceptible population. Mathematically, R_0_ is defined by the product of transmission rate and infectious period, both of which are dependent on pathogenicity and transmissibility (i.e., the intrinsic ability of the virus to be transmitted from one individual to another) [14]. Consequently, tissue tropism–which contributes to determining pathogenicity and transmissibility–will be under strong selective pressure to maximize fitness. In other words, there may be an optimal location for influenza virus infection along the respiratory tract that maximizes R_0_ [15]. However, the relationships between influenza virus tissue tropism in individual hosts and reproductive fitness at the population level are currently poorly understood, hence the selective pressures on influenza virus tissue tropism are not well characterized.

In this paper, we use cross-scale mathematical models of infection dynamics linking influenza virus within-host dynamics in a spatially-structured respiratory tract to population-level dynamics of transmission in a homogeneous and well-mixed population, to unveil the possible selective pressures on influenza virus tissue tropism. The proposed framework builds on recent developments in the cross-scale modeling of virus infection dynamics, whereby parameters of between-host models are estimated based on the dynamics of infection in individual hosts, as captured by within-host models [15]–[17]. These cross-scale or nested models have shed light on the evolutionary dynamics of immune escape and virulence by linking within-host and population-level scales; dynamics that could not be revealed by models addressing either of these scales separately. We propose to use a similar approach to explore the evolutionary dynamics of influenza virus tissue tropism. Because tissue tropism specifically refers to spatially-explicit dynamics within individual hosts, we introduce spatially-structured within-host models into the framework. Spatially-structured within-host models have proved useful tools for the study of within-host infection dynamics of human immunodeficient virus and hepatitis C virus in the presence of target cell heterogeneities [18], [19]. They shed light on the mechanisms behind multiple phases of infection or different courses of disease progression. Here we show that spatially-structured within-host models nested into between-host models of infection dynamics can provide insights into possible selective pressures shaping influenza virus tissue tropism, and highlight the reciprocal relationships between influenza virus tissue tropism within hosts and reproductive fitness at the population level.

## Materials and Methods

### Conceptual Overview

**Figure 1:**
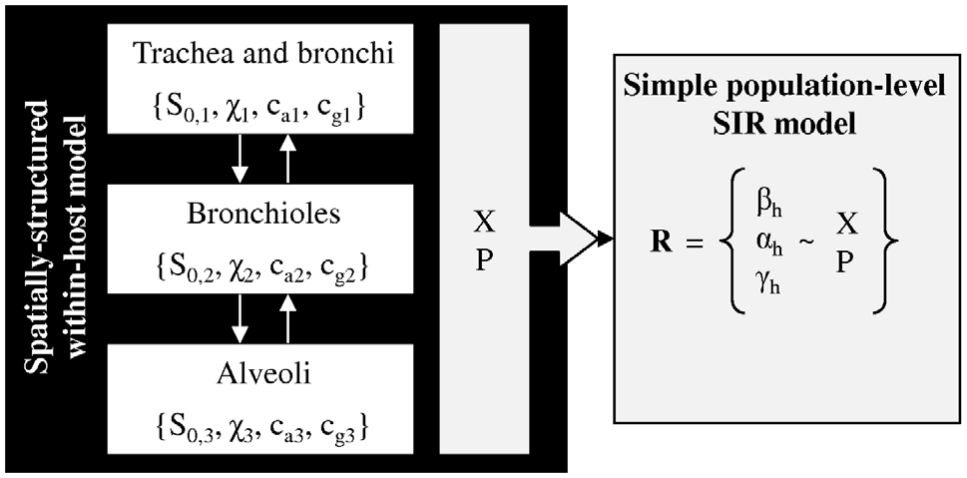
Conceptual overview of the framework. Within-host models of infection dynamics in a spatially-structured respiratory tract composed of three linearly-connected compartments are used to estimate overall virus excretion (X) and pathogenicity (P). The parameters that differ per respiratory compartment (i) are the initial number of susceptible cells (S_0,i_), the viral clearance rate (χ_i_), and the distribution coefficients of immunoglobulins of type A (IgA) and IgG (c_ai_ and c_gi_, respectively). The measures of virus excretion (X) and pathogenicity (P) in turn are used to estimate population-level transmission rate (β_h_), mortality rate (α_h_), and recovery rate (γ_h_), which define the virus reproductive number (R). See Methods for more details.

Within-host models of infection dynamics in a spatially-structured respiratory tract composed of linearly-connected respiratory compartments were developed. These allowed to quantitatively assess overall viral excretion (X) and pathogenicity (P) of viruses with different cellular tropisms. The quantitative measures of viral excretion (X) and pathogenicity (P) were then used to estimate the population-level reproductive number (R) of the viruses when circulating in a homogeneous and well-mixed human population (Fig. 1). Optimal tissue tropisms were defined as the cellular tropisms that maximized the virus reproductive number.

### Mathematical Model of Within-host Infection Dynamics

We developed a set of coupled differential equation models to capture the dynamics of influenza virus infection and host immune responses in the respiratory tract of individual hosts. Host adaptive immune responses were modeled in order to allow re-infection of hosts with pre-existing immunity. The minimal general format of the models was based on previously published work [16], [17], [20]–[22], modified as follows:

Susceptible cells 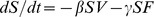



Infected cells 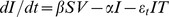



Cells refractory to infection 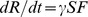



Free infective virus
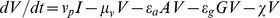



Type I interferon 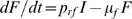



Cytotoxic T cells 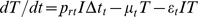



IgA 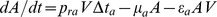



IgG 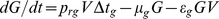
(1)


Susceptible cells (S) become infected with virus (V) at an infectivity rate (β) or become refractory to infection due to the action of type I interferon (F) at a rate γ. A refractory state to infection, triggered by host innate immune responses (type I interferon), was included to avoid cell depletion [20]. Infected cells (I) are killed by the infection at a rate α and by cytotoxic T cells (T) at a rate ε_t_. They produce virus (V) at a rate v_p_. Virus (V) decays at a rate µ_v_, and is neutralized by immunoglobulins of type A (IgA; A) at a rate ε_a_ and by immunoglobulins of type G (IgG; G) at a rate ε_g_, or is cleared by the muco-ciliary escalator at a rate χ. Type I interferon (F) is produced proportionally to the number of infected cells (I) at a rate p_rf_ and decays at a rate µ_f_. Cytotoxic T cells (T) proliferate proportionally to the number of infected cells (I) at a rate p_rt_, with a delay of Δt_t_, and die at a rate µ_t_. IgA (A) and IgG (G) are produced proportionally to the number of free virus (V) at a rate p_ra_ and p_rg_, with a delay of Δt_a_ and Δt_g_, and decay at a rate µ_a_ and µ_g_, respectively. IgA and IgG levels one year following primary infection are used as pre-existing immunity levels to model re-infection dynamics.

More complex versions of the models included the following variations. Birth and natural death processes of respiratory epithelial cells were added. The birth rate of respiratory epithelial cells was set based on the initial appearance of epithelial hyperplasia following infection, which typically appears along the respiratory tract around 3 days post-infection, irrespective of the region involved [3], [23]. The death rates of respiratory epithelial cells along the airways and of pneumocytes in the alveoli were set based on their respective lifespan of 6 and 18 months [24]. The immune system was further modeled with variable complexity, to include in most complex versions, the coupled dynamics of antigen-presenting cells, natural killer (NK) cells, T-helper cells, plasma B cells, and cytotoxic T lymphocytes, based on previous models [21], [25]. Lastly, the intermediary state of exposed cells, characterizing infected-non-infectious cells, was added in both simple and complex versions of the model to obtain SEI-type models [20].

#### Spatially-structured respiratory tract

The tracheo-bronchial tree is composed of the trachea and successive generations of bifurcating bronchi and bronchioles of decreasing length and diameter, through which air is transported. Terminal and respiratory bronchioles feed into alveolar ducts and inter-connected alveoli forming pulmonary acini, where vital gas exchange takes place. The bifurcating structure of the respiratory tract allows for a rapidly expanding epithelial surface area (and thus cell pool) towards the deeper lungs [26] (Table 1). Different cell types line different parts of the respiratory tract [26]–[28]. The tracheal and bronchial epithelium contains predominantly ciliated cells, as well as mucus-producing goblet cells. Together, these cells form the muco-ciliary escalator, which traps and propels particles towards the pharynx [29]. In contrast, the bronchiolar epithelium contains predominantly non-ciliated cells. The alveolar epithelium is composed of flat type I pneumocytes, forming the thin barrier between air and blood, and more numerous cuboidal type II pneumocytes, secreting surfactant and reabsorbing fluids [3].

**Table 1:** Characteristics of the human respiratory tract.

	Trachea and bronchi	Bronchioles	Alveoli	Ref.
**Epithelial surface area**	∼ handkerchief		> half a tennis court	[26]
**Main cell types (type of sialic** **acid-galactose linkage)**	Ciliated epithelial cells (α2,6)and goblet cells (α2,3)	Non-ciliated epithelialcells (α2,3)	Type I pneumocytes (α2,6) andtype II pneumocytes (α2,3)	[4]–[7], [26]–[28], [34]
**Particle clearance within** **24 hours**	High	Intermediate	Low	[29]–[32]
**Severity of damage** **(resulting disease)**	Mild (tracheo–bronchitis)	Moderate (bronchiolitis)	Severe (pneumonia)	[3], [67]
**Concentration of IgA**	High	Intermediate	None	[36], [37]
**Concentration of IgG**	Low	Intermediate	High	[36], [37]

IgA: immunoglobulins of type A; IgG: immunoglobulins of type G.

Heterogeneities in the abundance, distribution and function of respiratory epithelial cells have a major impact on different properties of the respiratory tract (Table 1). First, because of the muco-ciliary escalator, particles depositing in the trachea and bronchi have a higher probability of being cleared towards the pharyngeal area than those depositing in the bronchioles and alveoli [29]–[33]. Second, because of the more delicate nature and vital function of deeper airways and alveoli, damage caused to the bronchiolar and alveolar epithelia has more severe consequences than damage to the tracheal and bronchial epithelia [3]. Third, different types of cellular receptors used by influenza virus are expressed on the surface of the different respiratory epithelial cells. Ciliated epithelial cells and type I pneumocytes predominantly harbor sialic acids with α2,6 linkage to galactose, which are the cellular receptors preferentially used by human influenza A viruses. In contrast, non-ciliated epithelial cells and type II pneumocytes predominantly harbor sialic acids with α2,3 linkage to galactose, which are the receptors preferentially used by avian influenza viruses [5]–[7], [34]. The cellular tropism of avian influenza viruses and the spatial distribution of their target cells, more abundantly present in DRRT in humans, explain at least partly their higher pathogenicity compared to that of human influenza viruses [5]–[7].

Lastly, the dynamics and nature of host immune responses markedly differ along the respiratory tract (Table 1). Following infection, specific neutralizing immunoglobulins of type A and G (IgA and IgG, respectively) are differently distributed along the respiratory epithelium, limiting re-infection of underlying cells [35]–[37]. IgA are actively secreted along the epithelium of the airways, with higher concentrations in URRT. They are short-lived and undetectable generally 1 to 3 months following infection. In contrast, IgG passively diffuse from the serum and mostly line the respiratory epithelium of DRRT. They are long-lived and can be detected in circulation several years after infection. Although cellular adaptive immune responses, including cytotoxic T-cell responses, have not been quantified in similar detail in different regions of the respiratory tract, they also appear stronger and longer-lived in DRRT [38], [39]. As a result, protection against re-infection with influenza virus typically lasts longer in DRRT than in URRT, preventing severe disease (associated with DRRT infection) but not necessarily infection (of URRT in particular).

The respiratory epithelium therefore is not a continuous and homogeneous cellular layer available for influenza virus infection, despite being a common assumption of most previously published within-host models of influenza virus infection dynamics [20]–[22], [25], [40]–[46]. Spatial heterogeneities associated with the structure of the respiratory tract can have marked impact on viral excretion and infection severity [44]. To account for these spatial heterogeneities, we applied the mathematical models of within-host infection dynamics to linearly-connected respiratory compartments delineating three different regions: trachea and bronchi (compartment 1), bronchioles (compartment 2), and alveoli (compartment 3), associated with one pulmonary lobe. These three regions were chosen because they correspond to histologically distinct structures of the respiratory tract. We did not include the naso-pharyngeal cavity because we aimed at determining influenza virus optimal tropism in the respiratory tract located distally from the pharynx (commonly referred to as the lower respiratory tract). Because the gradients in cell numbers, clearance, pathogenicity and antibody concentrations follow the same trends when the naso-pharyngeal cavity is included [3], [26], [36], [37], adding this compartment to the model is unlikely to change the overall patterns described below.

To account for the differences in abundance and distribution of cells with sialic acids with α2,3 or α2,6 linkage to galactose, we considered two populations of cells in each respiratory compartment. Characteristics other than the linkage type of the sialic acids (e.g., shape and length as well as glycan modification and sialylation [47]–[49]) are undoubtedly important in defining the suitability of these as receptors for influenza virus. However, the distribution and abundance along the respiratory tract of the diversity of sialic acids beyond the linkage type have not been assessed in sufficient quantitative details for adequate modelling. Therefore, we assumed that the non-ciliated respiratory epithelial cells along the airways and type II pneumocytes in the alveoli harboured α2,3 receptors (recognized by avian influenza viruses), and that the ciliated respiratory epithelial cells along the airways and type I pneumocytes in the alveoli harboured α2,6 receptors (recognized by human influenza viruses), based on attachment pattern studies [4]–[6]. This assumption may lead to an overestimation of the number of susceptible cells available for infection; however, it captured the relevant trends in the relative abundance of susceptible cells available for avian or human influenza virus infection, as seen in attachment pattern studies [4]–[6]. Namely, the relative abundance of cells available for avian influenza virus infection increased deeper down the respiratory tract, while that of cells available for human influenza virus infection was larger in URRT. The infectivity rate (β) was modulated by affinity coefficients (a_2,3_ and a_2,6_) in the respective cell populations to account for variable receptor binding affinity profiles. The general format of the models based on two populations of susceptible cells and applied to a respiratory compartment (i) was defined as follows:

Cells with sialic acids with α2,3 linkage to galactose.

Susceptible cells 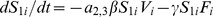



Infected cells 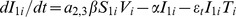



Cells refractory to infection 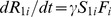
(2)


Cells with sialic acids with α2,6 linkage to galactose.

Susceptible cells 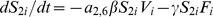



Infected cells 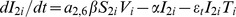



Cells refractory to infection 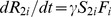
(3)


The dynamics of infection and host immune responses were linearly connected between the three respiratory compartments, as follows (for compartment i):

Total infected cells 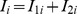



Free infective virus 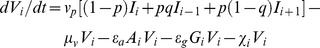



Type I interferon 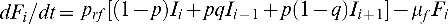



Cytotoxic T cells 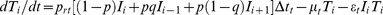
(4)


Free infective virus present in one compartment (V_i_) were produced by infected cells from the same compartment (I_i_) at a probability 1-p; from the compartment located above (I_i−1_) at a probability pq; and from the compartment located below (I_i+1_) at a probability p(1-q). Similarly, type I interferon present in one compartment (F_i_) was produced by infected cells from the same compartment (I_i_) at a probability 1-p; from the compartment located above (I_i−1_) at a probability pq; and from the compartment located below (I_i+1_) at a probability p(1-q). Lastly, cytotoxic T lymphocytes proliferating in one compartment (T_i_) were stimulated by infected cells from the same compartment (I_i_) at a probability 1-p; from the compartment located above (I_i−1_) at a probability pq; and from the compartment located below (I_i+1_) at a probability p(1-q). The quantities pq and p(1-q) were set proportional to the cross-sectional areas of the smallest bronchi and bronchioles, and ranged from 10^−3.5^ to 10^−1.8^.

All parameters of the within-host models of infection dynamics were identical in each respiratory compartment (i), except for the initial number of susceptible cells (S_i,0_) (and their death rate if included; see above), the rate of virus clearance (χ_i_) and the distribution coefficients of IgA and IgG (c_ai_ and c_gi_, respectively). The number of cells susceptible to infection in each respiratory compartment (S_0,i_) was estimated for the surface area of trachea and bronchi, bronchioles and alveoli associated with one pulmonary lobe, based on the Weibel model of the respiratory tract and published estimates of the proportions of the various cell types in different regions of the adult respiratory tract [26]–[28]. It resulted in 10^7^.^7^, 10^8^.^9^ and 10^10^.^2^ epithelial cells with sialic acids with α2,6 linkage to galactose; and 10^7^.^5^, 10^9^.^5^ and 10^10^.^5^ epithelial cells with sialic acids with α2,3 linkage to galactose in the trachea and bronchi, bronchioles, and alveoli, respectively. The virus clearance rate from each of the respiratory compartments (χ_i_) differed in accordance with data on the clearance rate of particles depositing in different regions of the respiratory tract [29]–[32]. It was estimated at 0.7 day^−1^ in the tracheo-bronchial compartment, 0.5 day^−1^ in the bronchiolar compartment and 0.05 day^−1^ in the alveolar compartment. Lastly, spatial heterogeneities in the distribution of IgA and IgG along the respiratory tract were introduced by adding distribution coefficients, c_ai_ and c_gi_, respectively, that differed per compartment. The distribution coefficients were estimated based on data obtained in mice [36], [37], because of the lack of data in humans (Table 2). We assumed that IgA and IgG were produced centrally upon infection before being redistributed in each compartment. This was done to mimic active excretion of IgA and passive diffusion of IgG from serum.

IgA 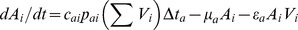



IgG 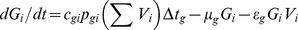
(5)


**Table 2:** Range of parameter estimates of the within-host models of infection dynamics.

	Parameter	Value	Ref.
β	Infectivity rate† (log)	−10.0 to −6.0	explored
γ	Refractory state rate (log)	−3 to −1.9	fitted
1/α	Lifespan of infected cells	12 hours	[80]
v_p_	Virus production rate (log)	2	fixed
1/µ_v_	Lifespan of virus particles	12 hours	[81]
κ	Viral clearance rate (day^−1^)	0.7 **(χ_1_)**; 0.5 **(χ_2_)**; 0.05 **(χ_3_)**	[29]–[32]
p_rf_	Production rate of type I interferon (log)	−1.9 to −0.9	fitted
p_rt_	Proliferation rate of cytotoxic T cells (log)	−3.1 to −2.5	fitted
p_ra_	Production rate of IgA (log)	−2.2 to 7.0	fitted/fixed‡
p_rg_	Production rate of IgG (log)	−3.1 to 7.0	fitted/fixed‡
c_a_	Distribution coefficient of IgA	0.7 **(c_a1_)**; 0.3 **(c_a2_)**; 0 **(c_a3_)**	[37]
c_g_	Distribution coefficient of IgG	0.01 **(c_g1_)**; 0.3 **(c_g2_)**; 0.7 **(c_g3_)**	[37]
1/µ_f_	Lifespan of type I interferon	8 hours	[82]
1/µ_t_	Lifespan of cytotoxic T cells	10 days	[83]
1/µ_a_	Lifespan of IgA	5 days	[84]–[86]
1/µ_g_	Lifespan of IgG	20 days	[84]–[86]
ε_t_	Killing rate of cytotoxic T cells (log)	−3.3 to −2.0	fitted
ε_a_	Neutralization rate of IgA (log)	−12.2 to −9.3	fitted
ε_g_	Neutralization rate of IgG (log)	−10.6 to −9.1	fitted

† Rates are given per hour if not otherwise indicated.

‡ In more complex versions of the models, IgA and IgG production rates were fixed to the maximal value indicated, to reduce the number of redundant free parameters.

The relative distribution of cytotoxic T cells in different regions of the respiratory tract is largely unknown therefore no spatial heterogeneities in their dynamics were introduced. When we retrospectively introduced higher production rates of cytotoxic T cells deeper down the respiratory tract, it did not alter the overall patterns described below (Fig. S3).

#### Fitting procedures

Although some parameters of the within-host models of infection dynamics could be derived from available data on human influenza virus infection, a number of parameters required estimation (Table 2). This was done by fitting the models by non-linear least squares to datasets of viral excretion in nasal washes [50], type I interferon concentration in nasal washes [51], IgA and IgG concentrations in serum [52] of human volunteers infected with seasonal influenza virus and, due to lack of human data, cytotoxic T lymphocyte (CD8+) counts in broncho-alveolar lavages of mice infected with seasonal influenza virus (mice being the most often used animal model of human immune response dynamics) [53], [54]. The sum of squared residuals (SSR) between the experimental data and the models’ results in log scale was minimized using the function *optim* in R [55]. Although the datasets used for the fitting procedures referred to samples collected from specific locations within the respiratory tract or from serum, we assumed that these data were representative of the dynamics of infection and immune responses in the respiratory tract as a whole; hence the curves produced by the models in each compartment were summed and fitted altogether to the data.

**Figure 2:**
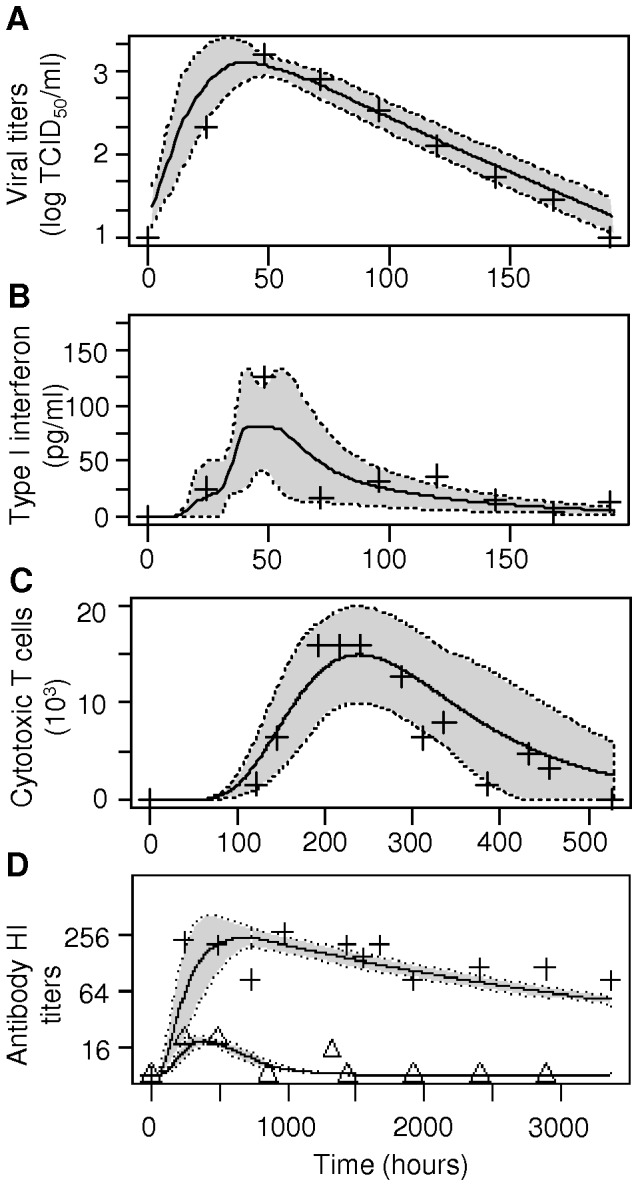
Within-host model dynamics. Output of within-host models is represented by curves. Black line: mean values across models; grey shaded area: standard deviation. Data points from empirical studies are represented by symbols. **A.** Viral shedding; **B.** Type I interferon; **C.** Cytotoxic T cells; **D.** Immunoglobulins of type A (IgA; triangles) and IgG (crosses). HI: hemagglutination inhibition.

Similarly to previously published models, simple and more complex versions of the within-host model of infection dynamics quantitatively captured the central features of the dynamics of viral infection and host immune responses (Fig. 2). However, we emphasize that some care must be taken when interpreting the individual parameter estimates because of possible redundancy among the parameters of simple and *a fortiori* more complex versions of the model. Nevertheless, uncertainty analyses on sets of estimated parameters using latin hypercube sampling [56] revealed that fitted estimates belonged to a robust parameter space that produced dynamics consistent with the available data (Fig. S1).

### Mathematical Model of Cross-scale Infection Dynamics

The within-host models of infection dynamics were subsequently nested into between-host models of the classic SIR (Susceptible-Infected-Recovered) type in a homogeneous and well-mixed human population:

Susceptible hosts 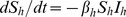



Infected hosts 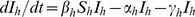



Recovered hosts 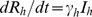
(6)


Susceptible hosts S_h_ become infected at a transmission rate β_h_. Infected hosts I_h_ die from infection at a mortality rate α_h_ or recover from infection at a recovery rate γ_h_.

#### Quantitative assessment of viral excretion and pathogenicity

 The transmission rate (β_h_), mortality rate (α_h_) and recovery rate (γ_h_) of the population-level SIR model were estimated based on quantitative assessment of viral excretion (X) and pathogenicity (P) derived from the within-host models of infection dynamics. Viral excretion (X) was approximated as the total amount of cleared virus above a transmission threshold (T), which was parameterized to obtain an infectious period (L_X_) within the range of published measures of influenza infectious period (3 to 5 days) [57]:
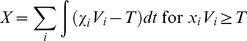
(7)


The use of a transmission threshold (T) allowed accounting for a minimal infectious dose below which virus transmission to a new individual was not successful.

We approximated pathogenicity (P) as the total number of infected cells (I) in the simple versions of the model: 
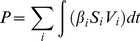
(8)


Because the lesions and clinical signs of influenza are a result of the death of respiratory epithelial cells, influx of inflammatory cells, and correlate with the levels of produced cytokines, we approximated pathogenicity (P) as the sum of the total number of infected cells, cytotoxic T lymphocytes and type I interferon in the more complex versions of the model; these sums were calculated over the duration of the infectious period (L_X_). X and P are correlates rather than explicit measures of viral excretion and pathogenicity, similarly to what can be measured empirically.

#### Estimation of SIR model transmission, mortality and recovery rates

Linear and non-linear (logistic) functions were explored to characterize the transmission and mortality rates (β_h_ and α_h_, respectively) as functions of viral excretion (X) and pathogenicity (P). The transmission rate (β_h_) was set as a positive function of viral excretion (X) and a negative function of pathogenicity (P). Pathogenicity (P) was used to estimate the transmission rate (β_h_) because lesions and clinical signs associated with influenza may on the one hand impede viral transmission (e.g., through damage to the muco-ciliary escalator, mechanical obstruction of the airways or both) and on the other hand impair infected individuals thereby reducing contacts between infected and non-infected individuals. The maximum value of β_h_ was fixed so that R_0_ reached a maximum between 2.5 and 3.5, in accordance with R_0_ estimates of pandemic influenza viruses in the human population [58], [59]. The mortality rate (α_h_) was set as a positive function of pathogenicity (P). The maximum value of α_h_ was fixed based on the case-fatality rate of highly pathogenic avian influenza H5N1, estimated at 60% [60]. The recovery rate (γ_h_) was set as the reciprocal of the duration of the infectious period (L_X_) when surviving infection: 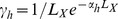
(9)


The actual relationships between viral excretion and pathogenicity on the one hand, and transmission, mortality and recovery rates of influenza on the other currently are poorly understood, and remain to be empirically characterized. Therefore, the framework described here is an exploration of the *possible* reciprocal feedbacks between tissue tropism and reproductive fitness, based on the general transmission-virulence trade-off hypothesis, i.e., on the assumption that a certain level of pathogenicity maximizes transmission [61]. As such, we assumed transmissibility was possible, regardless of receptor binding affinity profile, and sustained human-to-human transmission only occurred when the virus basic reproductive number was above one (R_0_>1; see below).

#### Calculation of influenza virus reproductive number

In an immunologically naïve population, the virus basic reproductive number (R_0_) was calculated as: 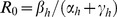
(10)


For R_0_>1, the effective reproductive number of the virus in a population with pre-existing immunity (R_e_) was calculated one year following virus circulation in an immunologically naïve population as: 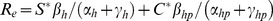
(11)where S* is the proportion of individuals immunologically naїve to the virus and equals 1/R_0_; and C* is the proportion of individuals with pre-existing immunity following infection the preceding year and equals 1–1/R_0_. The subscripts _h_ and _hp_ define rates following primary infection and re-infection with influenza virus, respectively, as determined by the measures of viral excretion (X) and pathogenicity (P) in a naїve individual and in an individual with pre-existing immunity, respectively. Contour plots of influenza virus reproductive number were drawn using lattice functions in R.

## Results

### Optimal Receptor Binding Affinity Profile

**Figure 3:**
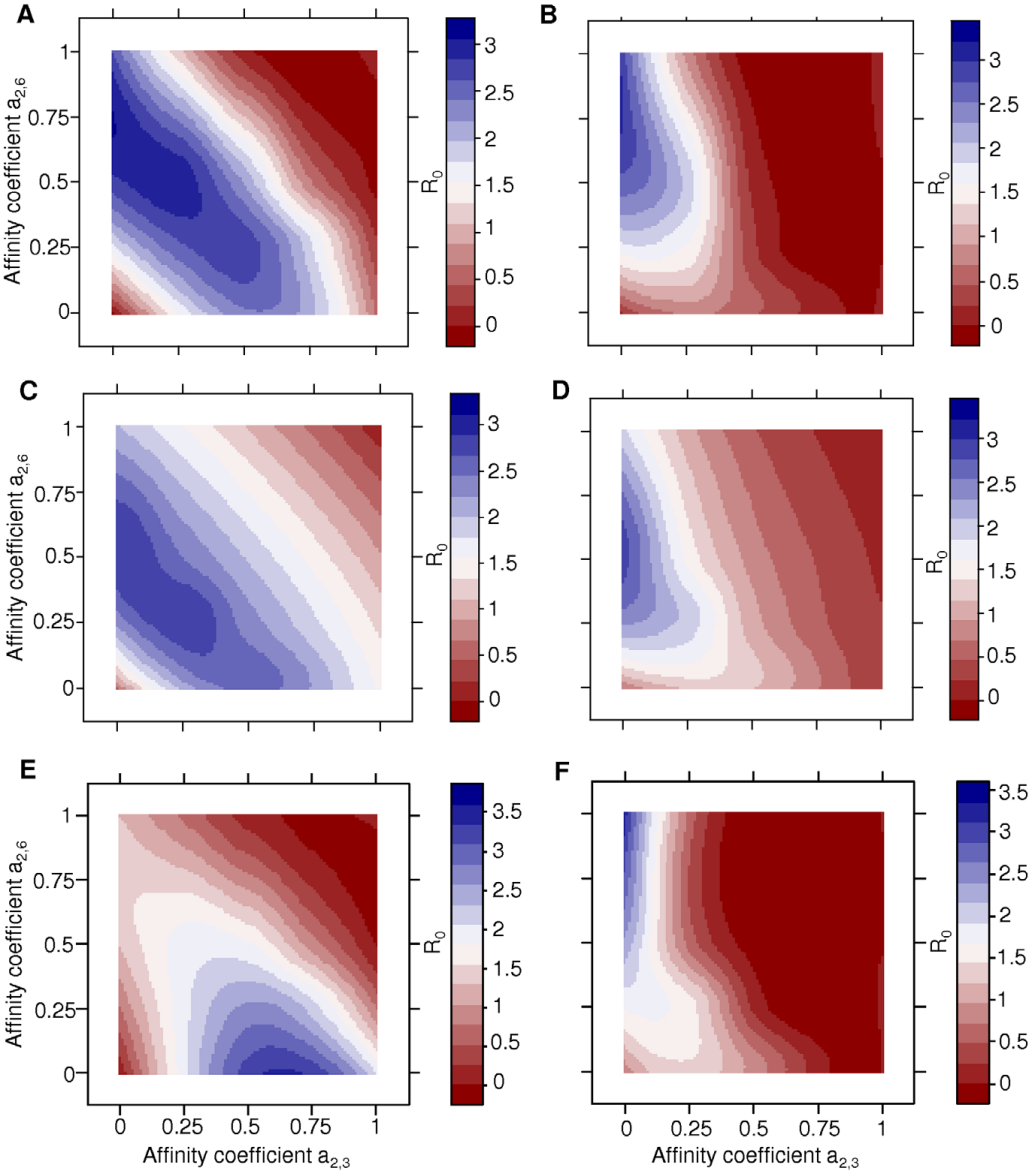
Optimal receptor binding affinity patterns. Contour plots of influenza virus basic reproductive number R_0_ (color scales) are drawn when the affinity coefficients a_2,3_ (x axis) and a_2,6_ (y axis) are varied from 0 to 1. For all graphs, the initial number of susceptible cells differ per respiratory compartment to reflect the heterogeneities in abundance and distribution of epithelial cells with sialic acids with α_2,3_ or α_2,6_ linkage to galactose. The effect of heterogeneities in viral clearance rates (χ_i_) and in the contribution of pathogenicity in each respiratory compartment (P_i_) to the overall virus pathogenicity (P) on the virus R_0_ is determined, when either non-linear or linear functions link within-host model output of viral excretion (X) and pathogenicity (P) to between-host model parameters. For panels **A** to **D**, χ_1_> χ_2_> χ_3_; for panels **E** and **F**, χ_1_ = χ_2_ = χ_3_. For panels **A**, **C** and **E**, P = ∑ P_i_; for panels **B**, **D** and **F**, P = P_1_+10^2^ P_2_+10^3^ P_3_. For panels **A**, **B**, **E** and **F**, non-linear functions link within-host model output to between-host model parameters; for panels **C** and **D**, linear functions link within-host model output to between-host model parameters.

We determined the optimal receptor binding affinity profile of influenza virus by varying the affinity coefficients a_2,3_ and a_2,6_ from 0 to 1. Influenza virus optimal receptor binding affinity profile was defined as the combination of affinity coefficients a_2,3_ and a_2,6_ that maximized the virus basic reproductive number (R_0_) in a human population with no pre-existing immunity. The framework predicted that a preferential affinity for sialic acids with α2,6 linkage to galactose (a_2,6_> a_2,3_) maximizes R_0_, and thus represents the optimal strategy for the sustained circulation of influenza virus in the human population (Fig. 3A). These results are in accordance with preferred α2,6 receptor binding affinity of human influenza viruses. However, there was a region in the parameter space of preferential α2,3 receptor binding affinity (a_2,3_> a_2,6_) that resulted in R_0_>1 (Fig. 3A).

In the above computation, virus pathogenicity (P) was calculated as the unweighed sum of pathogenicity (P_i_) in each respiratory compartment (i): 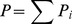
(12)


In other words, similar extent of damage caused to the epithelium in each respiratory compartment contributed equally to the overall pathogenicity of the infection (P). To further take into account the more delicate nature and vital function of bronchioles and alveoli, we alternatively calculated P as a weighed sum of P_i_ where the contribution of pathogenicity in the bronchiolar compartment (P_2_) and in the alveolar compartment (P_3_) was 10^2^ and 10^3^ times as much, respectively, as that in the tracheo-bronchial compartment (P_1_): 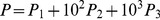
(13)


Under these conditions, preferential α2,6 receptor binding affinity maximized R_0_, as previously. However, preferential α2,3 receptor binding affinity generally resulted in R_0_<1 (Fig. 3B). This suggests that heterogeneities in the extent of damage associated with the infection in different regions of the respiratory tract may contribute to the limited ability of influenza virus with preferred α2,3 receptor binding affinity (e.g., avian influenza viruses) to efficiently circulate in the human population.

In the above computations, non-linear functions were used to link within-host model output of viral excretion (X) and pathogenicity (P) to between-host model transmission rate (β_h_), mortality rate (α_h_), and recovery rate (γ_h_). Similar optimal receptor binding affinity patterns were obtained when using linear functions (Fig. 3C and 3D). To determine whether these optimal patterns were driven solely by the differences in the number of susceptible cells in each respiratory compartment (S_0,i_), we set the virus clearance rates (χ_i_) or IgA and IgG distribution coefficients (c_ai_ and c_gi_) to identical values in all three respiratory compartments, in combination with equal or heterogeneous relative contributions of pathogenicity in each compartment. Setting virus clearance rates to identical values (χ_1_ = χ_2_ = χ_3_) resulted in opposite patterns depending on the relative contributions of pathogenicity in each compartment. When the relative contributions were equal (Eq. 12), a preferential α2,3 receptor binding affinity maximized R_0_ (Fig. 3E); in contrast, heterogeneities (Eq. 13) resulted in optimal α2,6 receptor binding affinity (Fig. 3F). On the other hand, setting IgA and IgG distribution coefficients to identical values (c_a1_ = c_a2_ = c_a3_ and c_g1_ = c_g2_ = c_g3_) did not alter the optimal receptor binding affinity patterns, regardless whether the relative contributions of pathogenicity in each compartment were equal or heterogeneous (data not shown). Therefore, the models suggest that spatial heterogeneities in virus clearance, virus pathogenicity or both may drive influenza virus towards a preference for α2,6 receptors.

### Optimal Infectivity Rates

Empirically, the tissue tropism of influenza viruses is not exclusively determined by their receptor binding affinity for sialic acids with α2,3 or α2,6 linkage to galactose. For example, sialic acid shape and length, as well as glycan modifications such as fucosylation, sulphation, and additional sialylation are known to modulate influenza virus binding affinity [47]–[49]. Furthermore, besides receptor binding affinity, variable replication efficiency can characterize related virus variants and result in different infection levels in different regions of the respiratory tract [62]–[64]. Accordingly, we explored the effect of variable infectivity for cells in the different regions of the respiratory tract on the reproductive fitness of human influenza virus (with affinity coefficients a_2,3_ = 0 and a_2,6_ = 1), by independently varying the infectivity rate (β_i_) in each respiratory compartment (i) from 10^−10^ to 10^−6^ h^−1^. Here, the maximum value of the mortality rate (α_h_) was based on the case-fatality rate of the 1918 pandemic influenza, estimated at 2% [2]. We determined the optimal tissue tropism of human influenza virus circulating in a completely naïve population (e.g., during a pandemic), as well as in a population with pre-existing immunity (e.g., during the following season). Optimal tissue tropism or optimal infectivity rates were defined as the combination of infectivity rates (β_i_) that maximized the virus reproductive number at the population level.

**Figure 4:**
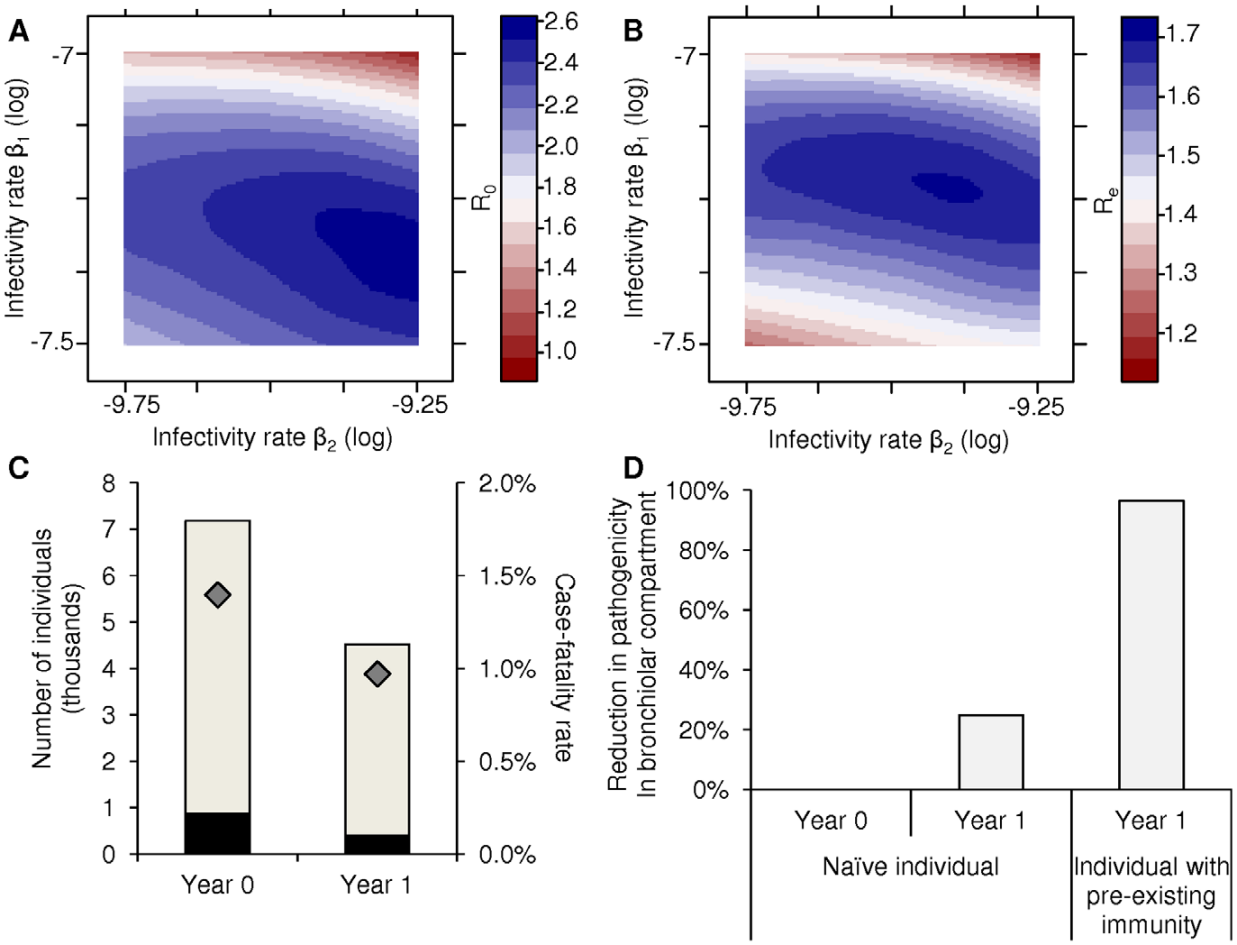
Optimal patterns of tissue tropism and associated morbidity and mortality burdens. Contour plots of influenza virus reproductive number (color scales) in an immunologically naïve population (R_0_; **A**) and in a partially-immune population (R_e_; **B**) are drawn when the infectivity rates β_2_ (x axis) and β_1_ (y axis) are varied. In all cases, the infectivity rate β_3_ is kept constant and equals the lowest infectivity rate in the explored range (10^−10^ h^−1^). Note that the optimal tissue tropism differs in an immunologically naïve and in a partially-immune population. **C.** The total number of cases per 10 000 individuals (light grey bars) and the number of fatal cases per 100 000 individuals (black bars) are represented for the influenza virus with optimal tissue tropism in an immunologically naïve population (year 0) and for the influenza virus with optimal tissue tropism in a partially-immune population (year 1). Their respective case-fatality rate is indicated by a dark grey diamond. **D.** The percentage reduction in pathogenicity in the bronchiolar compartment (P_2_) of the influenza virus with optimal tissue tropism in a partially-immune population is shown in a naïve individual and in an individual with pre-existing immunity in year 1 compared to that of the influenza virus with optimal tissue tropism in an immunologically naïve population (year 0).

**Figure 5:**
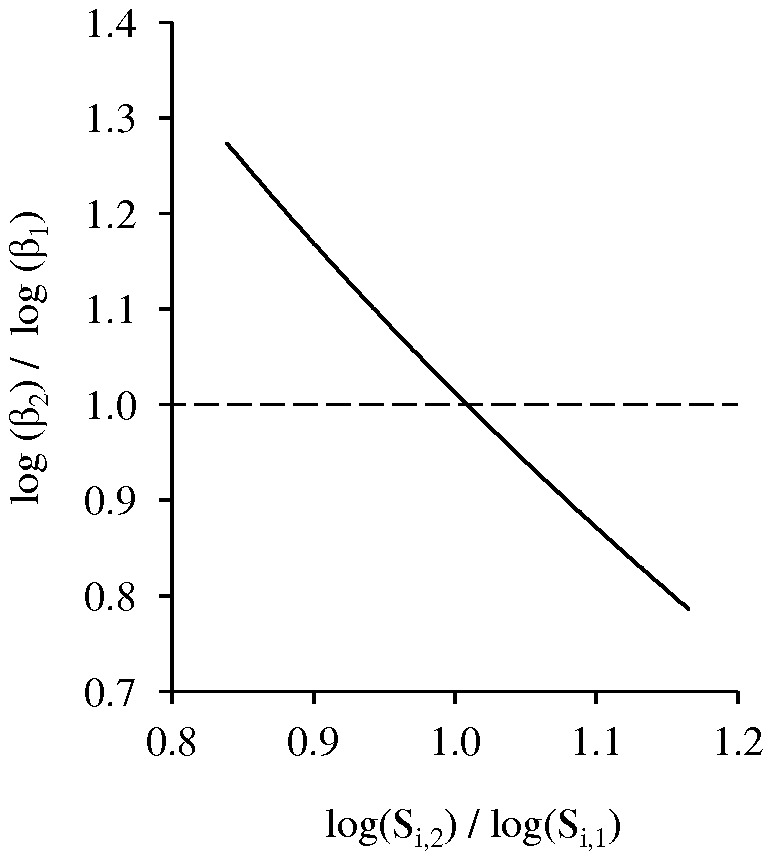
Relationship between optimal β_2_/β_1_ ratio and S_i,2_/S_i,1_ ratio in the absence of pre-existing immunity. The optimal infectivity rate in the bronchiolar compartment (β_2_) is smaller than the optimal infectivity rate in the tracheo-bronchial compartment (β_1_) provided that the initial number of susceptible cells in the bronchiolar compartment (S_i,2_) is larger than the initial number of susceptible cells in the tracheo-bronchial compartment (S_i,1_).

The optimal tissue tropism of human influenza virus in an immunologically naïve population was characterized by an infectivity rate in the tracheo-bronchial compartment (β_1_) that was approximately 100-fold greater than in the bronchiolar compartment (β_2_; Fig. 4A); the infectivity rate in the alveolar compartment (β_3_) that maximized R_0_ was consistently the lowest in the explored range (1000-fold lower than β_1_). The optimal β_2_/β_1_ ratio was maintained above 1 as long as the initial number of susceptible cells in the bronchiolar compartment (S_i,2_) was greater than that in the tracheo-bronchial compartment (S_i,1_; Fig. 5). These trends were obtained whether the relative contributions of pathogenicity in each compartment (P_i_) to overall pathogenicity (P) were equal or heterogeneous (Eq. 12 and 13, respectively). These results suggest that the heterogeneities in the abundance of target cells along the respiratory tract contribute to decreasing optimal infectivity rates of human influenza virus deeper down the respiratory tract.

The presence of pre-existing immunity changed the optimal tissue tropism of human influenza virus: optimal infectivity rate in the tracheo-bronchial compartment (β_1_) was higher, optimal infectivity rate in the bronchiolar compartment (β_2_) was lower, while optimal infectivity rate in the alveolar compartment (β_3_) remained lowest within the explored range (Fig. 4B). As expected, the presence of pre-existing immunity resulted in a decrease in the reproductive number of human influenza virus. The maximal values of R_e_ did not exceed 1.7 and were in the upper range of estimates of the reproductive number of seasonal influenza viruses [65]. The lower reproductive number, together with the changes in optimal tissue tropism, resulted in a decrease in both the infection rate and case-fatality rate in the partially immune population, compared to those in the immunologically-naïve population (Fig. 4C). The reduced case-fatality rate was caused in part by reduced pathogenicity in the bronchiolar compartment (P_2_) both in naïve and immune individuals (Fig. 4D); this was associated with the reduced optimal infectivity rate in the bronchiolar compartment (β_2_). The models thus indicate that influenza viruses circulating in partially-immune populations may have increased tropism for URRT and be less pathogenic than influenza viruses circulating in immunologically naïve populations, due to decreased tropism for DRRT.

### Sensitivity to Model Structure and Parameters

Similar tropism patterns (Fig. S2 and S3) were produced by both simple and more complex versions of the within-host model of infection dynamics, combining either linear or non-linear functions linking within-host model output of viral excretion (X) and pathogenicity (P) to between-host model transmission rate (β_h_), mortality rate (α_h_), and recovery rate (γ_h_). Sensitivity analyses to variations in the model parameters that were identical in all three respiratory compartments were performed. Latin hypercube sampling [56] was used to generate 100 sets of randomly sampled non-fitted parameter values within 50%–200% of their initial values, against which the estimated parameters were fitted by non-linear least squares. Of these 100 sets of parameters, 10 were randomly chosen to determine the tropism patterns of human influenza virus in an immunologically naïve and in a partially-immune population. For all models, the optimal infectivity rate in the tracheo-bronchial compartment (β_1_) was consistently higher when the virus was circulating in a partially-immune population than when it was circulating in a naïve population; conversely, the optimal infectivity rate in the bronchiolar compartment (β_2_) was consistently higher when the virus was circulating in a naïve population (Fig. S2). Therefore, the observed differences between the optimal tissue tropism of human influenza virus circulating in an immunological naïve population and that of human influenza virus circulating in a partially-immune population were not sensitive to the overall model structure and parameters common to all three respiratory compartments. We further evaluated the impact of each of the within-host model parameters that differed per respiratory compartment on these observed differences. Setting either the initial number of susceptible cells, the virus clearance rate, or IgA distribution coefficient in each compartment to equal values (S_0,1_ = S_0,2_ = S_0,3_, χ_1_ = χ_2_ = χ_3_, or c_a1_ = c_a2_ = c_a3_, respectively) did not alter the observed differences in optimal tissue tropism patterns in naïve and partially-immune populations (data not shown). In contrast, setting IgG production rate in each compartment to equal values (c_g1_ = c_g2_ = c_g3_) abolished these differences, and resulted in the same optimal infectivity rates in each compartment, independently of pre-existing immunity levels. This suggests that long-lived IgG present more abundantly in DRRT than in URRT following infection may drive influenza virus tissue tropism towards URRT in a human population with pre-existing immunity.

In conclusion, the observed optimal tropism patterns obtained in this study arose from the characteristic differences between the three respiratory compartments, and were independent of the structure or level of complexity of the within-host model of infection in each of these compartments. First, the optimal receptor binding affinity for sialic acids with α2,6 linkage to galactose was associated with differences in virus clearance or virus pathogenicity (or the interactions between both) in the three respiratory compartments. Second, the optimal infectivity rates in the three respiratory compartments, which exhibited a decreasing trend towards DRRT, were associated with the expanding cell pool deeper down the respiratory tract, in combination with lower clearance rates. Lastly, the increased URRT tropism and decreased DRRT tropism of influenza virus circulating in a population with pre-existing immunity were associated with spatial heterogeneities in the distribution and concentration of IgG in the three respiratory compartments.

## Discussion

The use of cross-scale models for influenza virus infection that link within-host dynamics in a spatially-structured respiratory tract to between-host dynamics at the population level, provides important insights into the possible selective pressures driving tissue tropism in the respiratory tract. To the best of our knowledge, these are the first such models developed at that interface. These models suggest that heterogeneities in virus clearance, virus pathogenicity, or the interactions between both, drive the receptor binding affinity of influenza virus towards sialic acids with α2,6 linkage to galactose. These models also indicate that the expanding cell pool deeper down the respiratory tract may lead to decreasing optimal infectivity rates towards DRRT, and that pre-existing immunity may drive influenza virus tissue tropism towards URRT.

The prediction of the cross-scale models that preferential α2,6 receptor binding affinity maximizes influenza virus reproductive fitness in a human population corresponds with field data, which show that both sporadic influenza pandemics and annual influenza epidemics are caused by influenza viruses with preferential α2,6 receptor binding affinity [2], [34], [66]. In the models, these patterns were driven by spatial heterogeneities in virus clearance or virus pathogenicity (or both) along the respiratory tract. Although the assumed heterogeneities in influenza virus clearance and pathogenicity associated with the location of infection currently cannot be quantified from empirical data, indications for these exist. The clearance of particles towards the pharyngeal area relies mainly on the muco-ciliary escalator, whereby mucus secreted by goblet cells and submucosal glands traps organic and inorganic particles before being propelled towards the pharynx by the movements of the cilia [29]. Extensive experimental studies have been performed on the clearance rates of magnetic or radio-labeled particles of variable size depositing in different regions of the respiratory tract of human volunteers (e.g., [29]–[32]). They revealed that the proportion of particles that are rapidly cleared towards the pharyngeal area decreases when they are deposited deeper down the respiratory tract. In particular, fast-cleared particles are mainly deposited along the larger airways. In contrast, the smaller airways are characterized by a higher proportion of slow-cleared particles, and most particles that deposit in the alveolar region are mainly cleared slowly by alveolar macrophages [33]. It is likely that virus particles produced in URRT are likewise being cleared towards the pharyngeal area at a higher rate than those produced in DRRT. As a consequence, the transmission of virus particles originating from URRT may be favored over that of virus particles originating from DRRT.

Spatial heterogeneities in pathogenicity associated with the location of influenza virus infection are well documented [3]. Primary viral pneumonia can occur upon acute influenza virus infection. It is a severe, if not fatal, respiratory disease resulting from infection of epithelial cells in DRRT and associated host immune responses. Because damage following DRRT infection affects vital gas exchange, it can result in respiratory insufficiency and death. In contrast, infection of URRT generally results in less severe disease, such as rhinitis and tracheo-bronchitis, associated with mild to moderate respiratory and general symptoms [3], [67]. Spatial heterogeneities in pathogenicity can result either from the higher number of infected cells in DRRT compared to that in URRT, due to the larger initial cell pool; or from damage that has relatively more severe consequences on the infected individual when located in DRRT than in URRT (i.e., when roughly the same number of cells are infected in both regions). We explored both assumptions by setting the relative contributions of pathogenicity in each respiratory compartment to either equal values or to heterogeneous values increasing deeper down the respiratory tract. Although the positive impact of coughing induced by URRT infection on transmission was not explicitly taken into account, heterogeneous contributions of pathogenicity increasing deeper down the respiratory tract would include such assumption. While the prediction of optimal receptor binding affinity for α2,6 receptors was maintained under both assumptions, the maintenance of R_0_ below 1 for viruses with preferred α2,3 receptor binding affinity was dependent on heterogeneous relative contributions of damage in each respiratory compartment to the overall pathogenicity of infection. Therefore, the increased pathogenicity of infection located deeper down the respiratory tract may contribute to the limited ability of influenza viruses with preferred α2,3 receptor binding affinity (e.g., avian influenza viruses) to efficiently circulate in the human population.

We further explored the optimal tissue tropism of human influenza viruses (with strict α2,6 receptor binding affinity) when circulating in a human population with or without pre-existing immunity. By allowing variable infectivity rates in the three respiratory compartments, the models predicted that progressive reduction of the virus infectivity for epithelial cells towards DRRT maximized influenza virus reproductive fitness. This pattern was associated with the expanding cell pool deeper down the respiratory tract, in association with decreasing clearance rates. The relative infection levels of different regions of the respiratory tract by influenza virus are not often reported in the literature. Because of the multifocal nature of influenza virus infection, it can be difficult empirically to quantitatively compare the proportion of infected cells in different regions of the respiratory tract. Nevertheless, the results of the models tend to be consistent with empirical data. In particular, seminal work by Smith and Sweet [63] clearly emphasized the heterogeneities in infection levels of various strains of influenza virus in URRT and DRRT of ferrets (considered the most appropriate animal model for influenza pathogenesis in humans [4]–[6]). Infection levels were typically found to be higher in URRT than in DRRT, and infection of alveolar epithelial cells was very rarely observed. In humans, infection is also typically more marked in URRT than in DRRT, and the absence of viral antigen in alveolar cells has also been noticed following fatal infection with human influenza virus [62], [65], [68], [69]. Among the factors potentially explaining low levels of influenza virus infection in the alveoli were lower viral production rates by alveolar epithelial cells [63]. Although the viral production rate was kept constant in the models, redundancy does occur between viral production rate and infectivity rate. Thus our findings are consistent with these observations, and may provide an insight into the evolutionary mechanism behind the lower infectivity of human influenza virus for DRRT.

Comparing the optimal tissue tropism of human influenza virus in an immunologically naïve population and in a population with pre-existing immunity indicated that pre-existing immunity may drive influenza virus tissue tropism even more towards URRT. Associated with this, the case-fatality rate of human influenza virus circulating in a partially immune population was reduced compared to that of human influenza virus circulating in an immunologically naïve population, in part due to reduced pathogenicity in DRRT. These results suggest that the tissue tropism of influenza viruses may vary according to pre-existing immunity levels in the human population. Low pre-existing immunity levels may result in the circulation of viruses with increased tropism for DRRT–and thus more pathogenic viruses–compared to that of viruses circulating in a population with high pre-existing immunity levels. This prediction is consistent with more frequent observation of infected cells in DRRT of naïve ferrets inoculated with pandemic influenza viruses than in DRRT of naïve ferrets inoculated with seasonal influenza viruses [64], [70].

Pre-existing immunity is also known to drive the evolution of immune escape variants of seasonal influenza virus. In particular, the surface proteins of seasonal influenza virus acquire amino-acid substitutions through genetic mutations that make them less recognizable by neutralizing antibodies targeting the surface proteins of preceding strains. Some mutations have greater antigenic effect than others [71], effectively reducing the level of pre-existing immunity. As a result, immune escape variants with large antigenic effect generally cause larger epidemics than do preceding strains [72], [73]. We may infer from our results that immune escape variants may also have higher tropism for DRRT than do preceding strains, and cause more severe disease. This would be the case if their antigenicity was sufficiently distant from previously-circulating strains that their circulation in the population would be comparable to that of a virus circulating in a naïve population. Although variations in the tissue tropism of seasonal influenza viruses in association with immune escape have not been studied in detail, this prediction is in accordance with increased DRRT tropism of a recent drift variant of seasonal influenza virus H3N2 compared to that of strains circulating the preceding years [62].

Because tissue tropism of influenza virus is among the phenotypic traits at the interface of within- and between-host infection dynamics, an individual host may not be considered as a black box with a homogeneous and continuous cell monolayer available for virus replication. Variations in tissue tropism may result in variations in transmission and infectious period, which are at the basis of the virus reproductive fitness, therefore creating reciprocal feedbacks between within- and between-host infection dynamics. The proposed cross-scale framework has shed light on potential selective pressures driving influenza virus tissue tropism along the respiratory tract, and has predicted patterns that are consistent with empirical observations. However, as with most models, there are a number of caveats. The framework builds on assumptions regarding the relationships connecting within-host infection dynamics and between-host transmission dynamics that currently cannot be verified due to lack of data. In particular, the models were built based on the general transmission-virulence trade-off hypothesis [61]. We assumed that disease negatively impacted virus transmission rate because it can impair infected individuals and therefore reduce their contact with other individuals; and also because respiratory lesions and associated inflammation may impair the muco-ciliary escalator and mechanically obstruct the airways, thus reducing excretion of virus particles. We propose that these relationships may be inferred by empirical testing of the predictions of this framework. Especially, the relationships between viral pathogenicity, excretion, and transmission in association with the location of infection along the respiratory tract may be characterized in animal models reproducing influenza virus pathogenesis as described in humans, such as the ferret. As part of the between-host component of the framework, only a well-mixed and homogeneous population of human hosts was considered, and heterogeneities in transmission rates and pre-existing immunity levels at the host population level were ignored. For example, it is generally recognized that school-age children play a major role in the transmission of influenza virus [74]–[77]. Furthermore, pre-existing immunity levels are strongly age-dependent [78], [79]. This calls for further development of the framework to include host population heterogeneities, in association with empirical testing of its predictions. Because of their very nature, even the simplest cross-scale models have a high number of free parameters, and their complexity can rapidly increase both at the within-host and between-host levels. However, sensitivity analyses and model comparisons demonstrated that the key fitness results arising from this study are robust. These key fitness results represent hypotheses that are testable in an empirical setting. As such, this model framework provides a template for the mechanistic study of influenza virus cross-scale evolutionary and epidemiological dynamics.

## Supporting Information

Figure S1 **Uncertainty analysis to simultaneous variations of within-host model parameter values.** **A**. Peak timing (days) and **B**. maxima (log) of viral excretion (1), type I interferon production (2), cytotoxic T cell proliferation (3), and immunoglobulins of type A (IgA; 4) and IgG (5) production, based on 1000 sets of parameters generated by latin hypercube sampling, ranging from 50% to 200% the fitted values. Thick line is the median value, box lower and upper limits are the second and fourth quartiles, and lower and upper whiskers are minimum and maximum values.
(DOC)

Figure S2 **Optimal tissue tropism of human influenza viruses in populations with different levels of pre-existing immunity based on simple and more complex versions of the models (including models with parameter sets generated by latin hypercube sampling; see text for details).** The maximal reproductive number of human influenza viruses is plotted for each value of their infectivity rate for (**A**) tracheal and bronchial, (**B**) bronchiolar, and (**C**) alveolar epithelial cells in a naïve population (black) and in a partially immune population (grey). Optimal tropisms are marked by a square. Infectivity rates are increasing from 1 to 5 as they differ per model. Standard errors are represented.
(DOC)

Figure S3 **Optimal patterns of tissue tropism based on more complex versions of the model.** Contour plots of influenza virus reproductive number (color scales) in an immunologically naïve population (R_0_; **left column**) and in a partially-immune population (R_e_; **right column**) are drawn when the infectivity rates β_2_ (x axis) and β_1_ (y axis) are varied. In all cases, the infectivity rate β_3_ is kept constant and equals the lowest infectivity rate in the explored range (10^−10^ h^−1^). **First row:** the within-host model of infection dynamics includes birth and death processes of respiratory epithelial cells. **Second row:** the within-host model of infection dynamics includes additional components of the immune response, namely antigen-presenting cells, NK cells, T-helper cells, plasma-B cells and CTL. CTL production rate increases deeper down the respiratory tract. **Third row:** the within-host model of infection dynamics includes an exposed state characterizing infected-non-infectious epithelial cells (SEI-type model). Infectivity rates increases from 1 to 5 as they differ per model.
(DOC)
