# Supplementary material for: Linking Influenza Virus Tissue Tropism to Population-Level Reproductive Fitness
Source: PLoS One. 2012 Aug 28;7(8):e43115. doi: 10.1371/journal.pone.0043115 (PMC3429484; doi:10.1371/journal.pone.0043115)
Supplement: Figure S1 — Uncertainty analysis to simultaneous variations of within-host model parameter values. A. Peak timing (days) and B. maxima (log) of viral excretion (1), type I interferon production (2), cytotoxic T cell proliferation (3), and immunoglobulins of type A (IgA; 4) and IgG (5) production, based on 1000 sets of parameters generated by latin hypercube sampling, ranging from 50% to 200% the fitted values. Thick line is the median value, box lower and upper limits are the second and fourth quartiles, and lower and upper whiskers are minimum and maximum values. (DOC) [file pone.0043115.s001.doc]

**Figure S1.** Uncertainty analysis to simultaneous variations of within-host model parameter values. **A**. Peak timing (days) and **B**. maxima (log) of viral excretion (1), type I interferon production (2), cytotoxic T cell proliferation (3), and immunoglobulins of type A (IgA; 4) and IgG (5) production, based on 1000 sets of parameters generated by latin hypercube sampling, ranging from 50% to 200% the fitted values. Thick line is the median value, box lower and upper limits are the second and fourth quartiles, and lower and upper whiskers are minimum and maximum values.

**
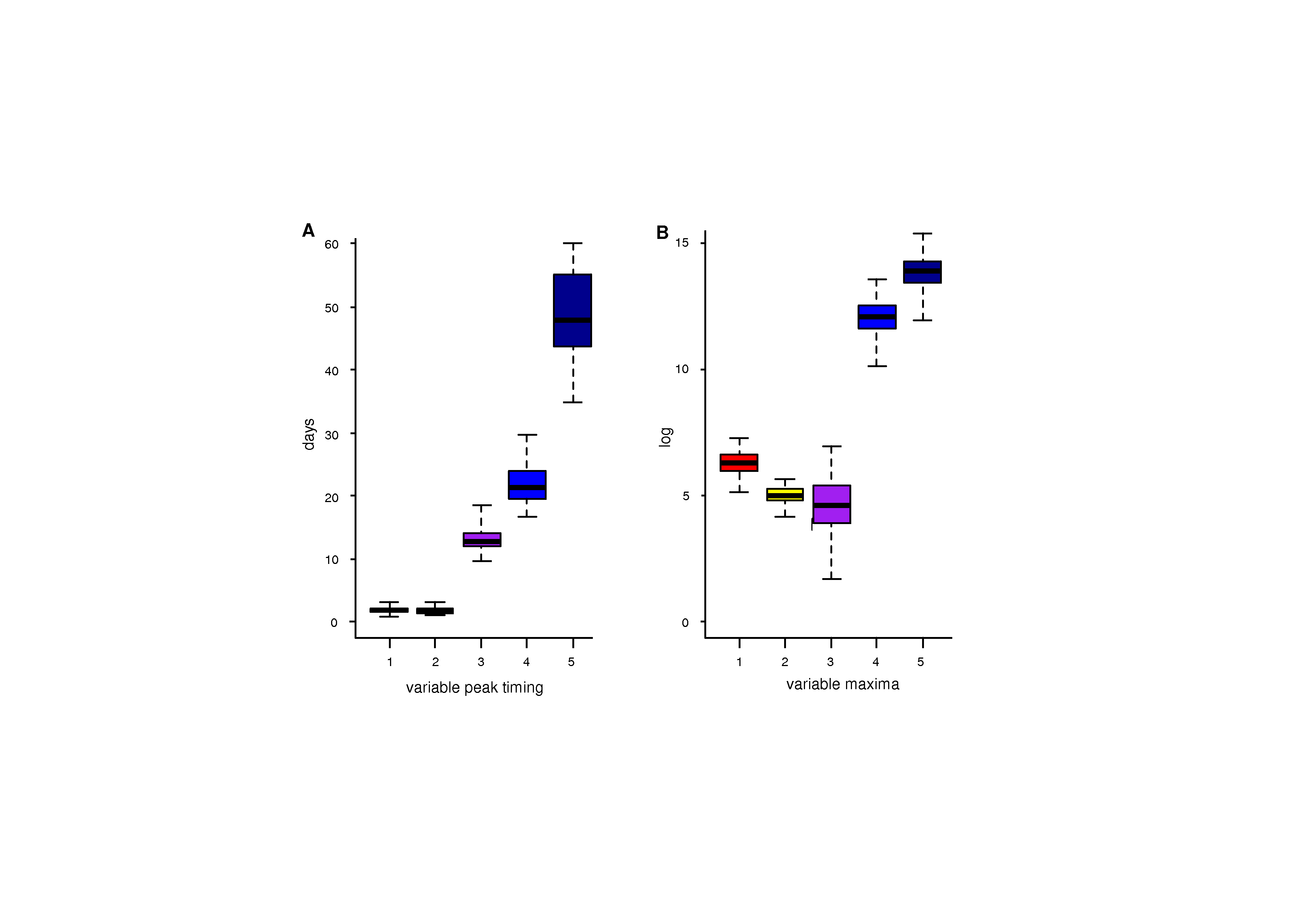
**
