# Supplementary material for: Linking Influenza Virus Tissue Tropism to Population-Level Reproductive Fitness
Source: PLoS One. 2012 Aug 28;7(8):e43115. doi: 10.1371/journal.pone.0043115 (PMC3429484; doi:10.1371/journal.pone.0043115)

**Figure S2.** Optimal tissue tropism of human influenza viruses in populations with different levels of pre-existing immunity based on simple and more complex versions of the models (including models with parameter sets generated by latin hypercube sampling; see text for details). The maximal reproductive number of human influenza viruses is plotted for each value of their infectivity rate for (**A**) tracheal and bronchial, (**B**) bronchiolar, and (**C**) alveolar epithelial cells in a naïve population (black) and in a partially immune population (grey). Optimal tropisms are marked by a square. Infectivity rates are increasing from 1 to 5 as they differ per model. Standard errors are represented.


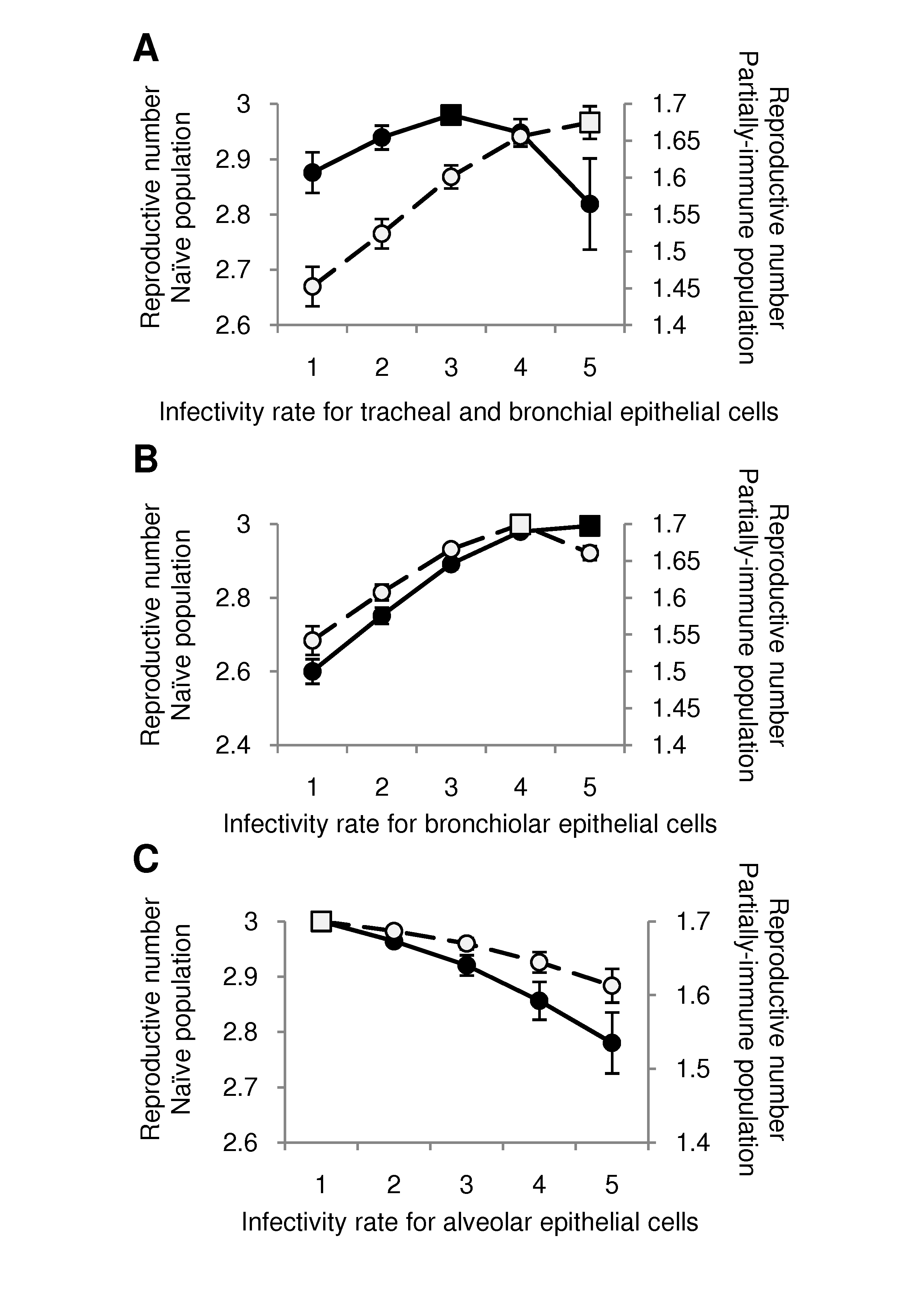

Supplement: Figure S2 — Optimal tissue tropism of human influenza viruses in populations with different levels of pre-existing immunity based on simple and more complex versions of the models (including models with parameter sets generated by latin hypercube sampling; see text for details). The maximal reproductive number of human influenza viruses is plotted for each value of their infectivity rate for (A) tracheal and bronchial, (B) bronchiolar, and (C) alveolar epithelial cells in a naïve population (black) and in a partially immune population (grey). Optimal tropisms are marked by a square. Infectivity rates are increasing from 1 to 5 as they differ per model. Standard errors are represented. (DOC) [file pone.0043115.s002.doc]
