# Supplementary material for: Linking Influenza Virus Tissue Tropism to Population-Level Reproductive Fitness
Source: PLoS One. 2012 Aug 28;7(8):e43115. doi: 10.1371/journal.pone.0043115 (PMC3429484; doi:10.1371/journal.pone.0043115)

**Figure S3.** Optimal patterns of tissue tropism based on more complex versions of the model. Contour plots of influenza virus reproductive number (color scales) in an immunologically naïve population (R_0_; **left column**) and in a partially-immune population (R_e_; **right column**) are drawn when the infectivity rates β_2_ (x axis) and β_1_ (y axis) are varied. In all cases, the infectivity rate β_3_ is kept constant and equals the lowest infectivity rate in the explored range (10^-10^ h^-1^). **First row:** the within-host model of infection dynamics includes birth and death processes of respiratory epithelial cells. **Second row:** the within-host model of infection dynamics includes additional components of the immune response, namely antigen-presenting cells, NK cells, T-helper cells, plasma-B cells and CTL. CTL production rate increases deeper down the respiratory tract. **Third row:** the within-host model of infection dynamics includes an exposed state characterizing infected-non-infectious epithelial cells (SEI-type model). Infectivity rates increases from 1 to 5 as they differ per model.


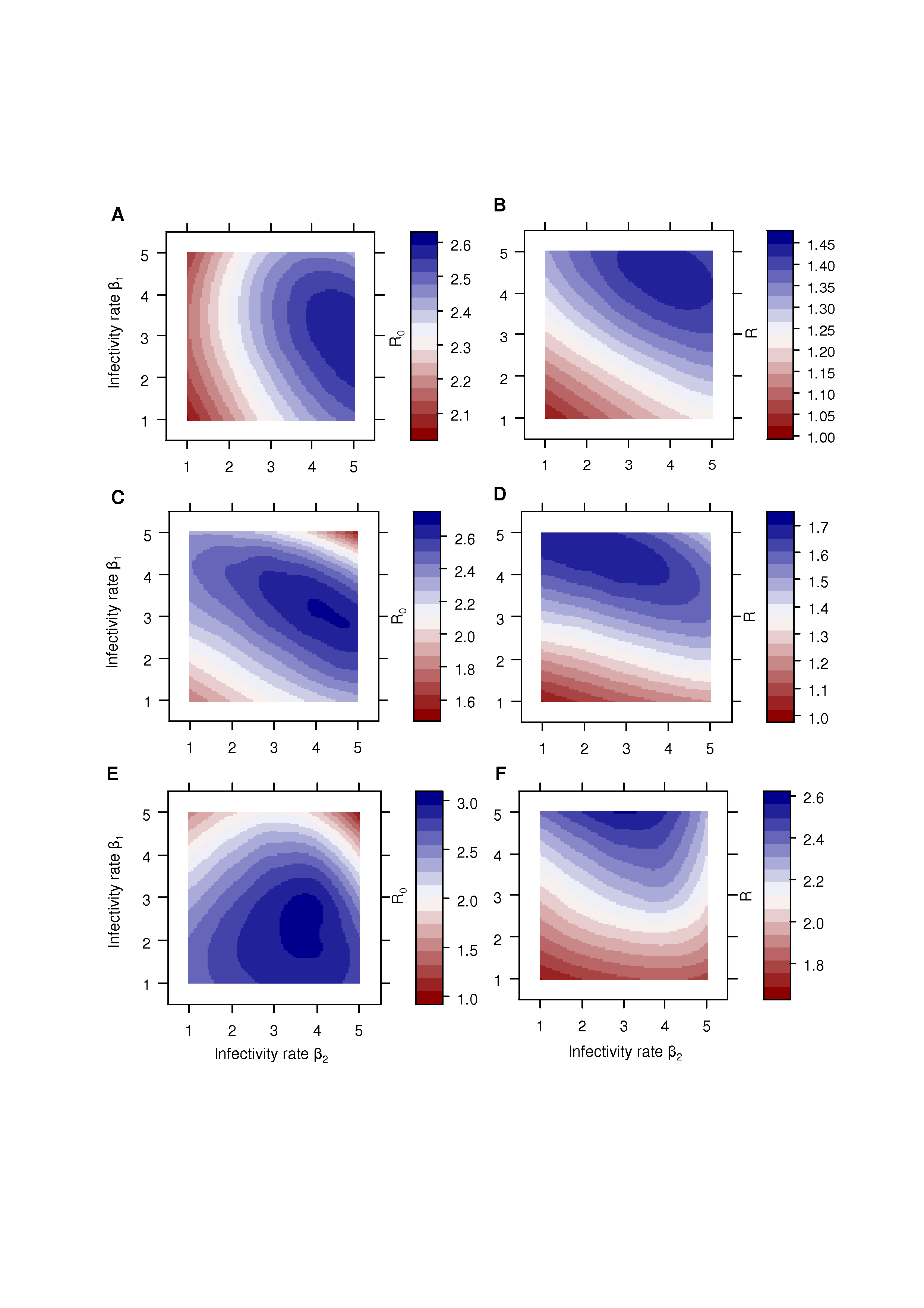

Supplement: Figure S3 — Optimal patterns of tissue tropism based on more complex versions of the model. Contour plots of influenza virus reproductive number (color scales) in an immunologically naïve population (R0; left column) and in a partially-immune population (Re; right column) are drawn when the infectivity rates β2 (x axis) and β1 (y axis) are varied. In all cases, the infectivity rate β3 is kept constant and equals the lowest infectivity rate in the explored range (10−10 h−1). First row: the within-host model of infection dynamics includes birth and death processes of respiratory epithelial cells. Second row: the within-host model of infection dynamics includes additional components of the immune response, namely antigen-presenting cells, NK cells, T-helper cells, plasma-B cells and CTL. CTL production rate increases deeper down the respiratory tract. Third row: the within-host model of infection dynamics includes an exposed state characterizing infected-non-infectious epithelial cells (SEI-type model). Infectivity rates increases from 1 to 5 as they differ per model. (DOC) [file pone.0043115.s003.doc]
